# What is polypharmacy in people living with HIV/AIDS? A systematic review

**DOI:** 10.1186/s12981-022-00461-4

**Published:** 2022-08-02

**Authors:** Mohammed Ibn-Mas’ud Danjuma, Safah Khan, Farah Wahbeh, Lina Mohammad Naseralallah, Unwam E. Jumbo, Abdelnaser Elzouki

**Affiliations:** 1 Division of General Internal Medicine, Weill Cornell Affiliated-Hamad General Hospital, Hamad Medical Corporation, Doha, Qatar; 2 College of Medicine, QU Health, Qatar University, Doha, Qatar; 3 Weill Cornell College of Medicine, New York and Doha, Qatar; 4 Clinical Pharmacy Department, Hamad Medical Corporation, Doha, Qatar; 5 School of Pharmacy, College of Medical and Dental Sciences, University of Birmingham, Birmingham, UK

**Keywords:** PLWHA, Polypharmacy, Drug interactions, HIV, AIDS, Antiretroviral

## Abstract

Polypharmacy in people living with HIV/AIDS (PLWHA) is a rising morbidity that exacts hefty economic burden on health budgets in addition to other adverse clinical outcomes. Despite recent advances, uncertainty remains around its exact definition in PLWHA. In this systematic review and Meta-analysis, we explored relevant databases (PUBMED, EMBASE, CROI) for studies evaluating polypharmacy in PLWHA from January 2000 to August 2021 to ascertain the exact numerical threshold that defines this morbidity. Two independent reviewers extracted and reviewed relevant variables for analyses. The review included a total of 31 studies involving n = 53,347 participants with a mean age of 49.5 (SD ± 17.0) years. There was a total of 36 definitions, with 93.5% defining polypharmacy as the concomitant use of 5 or more medications. We found significant variation in the numerical definition of polypharmacy, with studies reporting it as “minor” (N = 3); “major” (N = 29); “severe” (N = 2); “excessive” (N = 1); and “higher” (N = 1). Most studies did not incorporate a duration (84%) in their definition and excluded ART medications (67.7%). A plurality of studies in PLWHA have established that polypharmacy in this cohort of patients is the intake of ≥ 5 medications (including both ART and non-ART). To standardize the approach to addressing this rising morbidity, we recommend incorporation of this definition into national and international PLWHA treatment guidelines.

**Supplementary Information:**

The online version contains supplementary material available at 10.1186/s12981-022-00461-4.

## Introduction

One of the evolving challenges with regards to therapeutics in people living with HIV/AIDS (PLWHA) is the increasing number of daily medications patients often must take [1–3]. This is invariably a consequence of the rising multimorbidity associated with increasing survival seen in these patient cohorts as well as those of the general population [4]. The latter although attributable to multiple factors, the role of antiretroviral therapy (ART) drugs is by far the most important on reducing mortality of PLWHA [3]. Unfortunately, since the incorporation of these drugs into national and international treatment guidelines, the total number of HIV and non-HIV medications used by PLWHA daily has exponentially increased. This has conferred enormous burden on this cohort of patients [5], including the costly consequences of polypharmacy such as drug–drug [6, 7], drug–food [8], and pharmacogenetic interactions [9].

In the general population, polypharmacy has often been defined as the daily ingestion of five or more medications [10]. Whilst there appears to be consensus regarding the definition of polypharmacy in the general population [10], uncertainty still exists as to what exactly constitutes polypharmacy in PLWHA [11]. This uncertainty revolves around both the numerical threshold (< 5, ≥ 5, or ≥ 10 medications etc.) [11], as well as whether HIV medications [12] were part of the numerical count of polypharmacy or not. Whilst several reports have attempted to explore patterns, determinants, and consequences of polypharmacy across various populations in both inpatient and outpatient settings around the world [8, 13–15], an enduring consensus around its exact definition in PLWHA remains unexplored. The most recent attempt at a numerical characterization of polypharmacy in this cohort of patients was a narrative review by Back et al. [1]. A recent meta-analysis by Danjuma et al. explored for the first time the prevalence as well as global trends of polypharmacy across different demographic populations of PLWHA (in press). It reported a period prevalent rate of polypharmacy amongst PLWHA of around 33% across the world and rising. Notably most of the studies included in this review synthesis had different medication thresholds for what constitutes polypharmacy.

In this study, we aimed to carry out a comprehensive synthesis of all studies that have investigated polypharmacy in PLWHA with the view to ascertaining what exactly constitutes polypharmacy in this cohort of patients; and in so doing engender potentially useful prescriptive consensus around this rising morbidity.

## Method

This systematic review followed the Preferred Reporting Items for Systematic reviews and Meta-Analyses (PRISMA) checklist and the Cochrane Handbook guidelines [16]. The protocol was registered on the International Prospective Register of Systematic Reviews (PROSPERO)—CRD42020170071.

### Eligibility criteria

The following databases were searched from 1 January 2000 to 30 August 2021: PubMed; EMBASE, Conference on Retroviruses and Opportunistic Infections (CROI), Cochrane Database of Systematic Reviews; Science Citation Index and Database of Abstracts of Reviews of Effects (DARE). Reference lists of included studies were also manually searched to identify relevant articles that were not yielded from the database search. Databases were searched using the Boolean operator ‘AND’ to combine terms from different categories, while ‘OR’ was utilized for terms under one category. The following Medical Subject Headings (MeSH) terms and keywords were used: (HIV [tiab] OR “people living with HIV” [MeSH] AND polypharmacy[tiab]). Studies were considered for inclusion if they were published in English language between 1st January 2000 and 30th august 2021; and focused on PLWHA with age greater than 18 years old. All studies that incorporated at least one definition (numerical, descriptive or both) of polypharmacy amongst PLWHA on ART medicines were eligible for inclusion irrespective of design. We excluded studies that failed to clearly define what constitutes polypharmacy either numerically or descriptively in their methodology. Additional file 1: Table S2 shows results of literature search.

### Study selection

Following completion of literature search from relevant databases, duplicates were removed utilizing EndNote 20^®^ (2021 Clarivate). Screening of titles, abstracts (using Rayyan QCRI software), and full papers (using Microsoft excel) was conducted independently by two reviewers (SK and FA) according to the inclusion and exclusion criteria. Discrepancies were resolved through consensus or by adjudication by a third reviewer (MID).

### Risk of bias assessment

Two reviewers (MID, LMN) carried out a comprehensive risk of bias assessment of the reviewed studies using the Cochrane checklist for the assessment of risk of bias [17]. Where disagreement arose, this was resolved with consensus or adjudication by the third reviewer (AE). This is to ascertain the methodological quality of the reviewed studies. Details of this checklist has exhaustively been explained elsewhere, but in brief it is comprised of six standard criteria: including concealment of allocation, adequate sequence generation, blinding of participants and personnel, from selective reporting, blinded assessment of primary outcome(s), freedom from other risks of bias, and adequately addressed incomplete outcome data. A risk of bias table showing the grading of the studies is shown in Additional file 1: Table S1.

### Data extraction

A data extraction form was designed by two reviewers (MID and SK) and piloted on five included studies. We extracted the following variables from each study: first author, year of publication, center where the study was carried out, study design, duration of HIV/AIDS, HIV viral load (where available), number of PLWHA, polypharmacy definition, number of patients satisfying criteria for polypharmacy, socio-demographic parameters. Where studies explored different definitions of polypharmacy, we included all definitions they explored. Statistical analyses were conducted in Stata Statistical Software: Release 17. College Station, TX: StataCorp LLC. 2021.

## Results

**Fig. 1:**
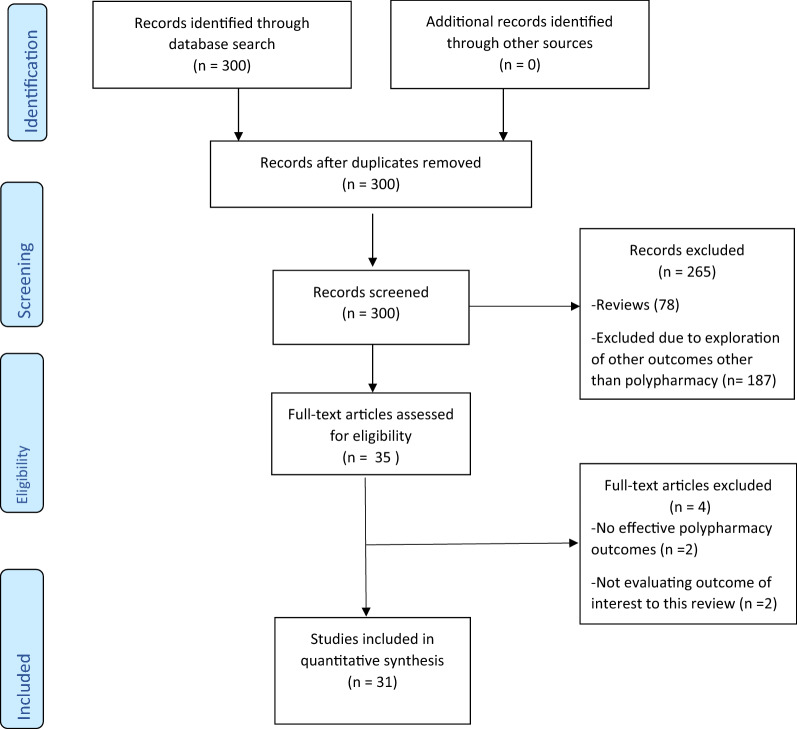
PRISMA flow chart for study selection

**Fig. 2:**
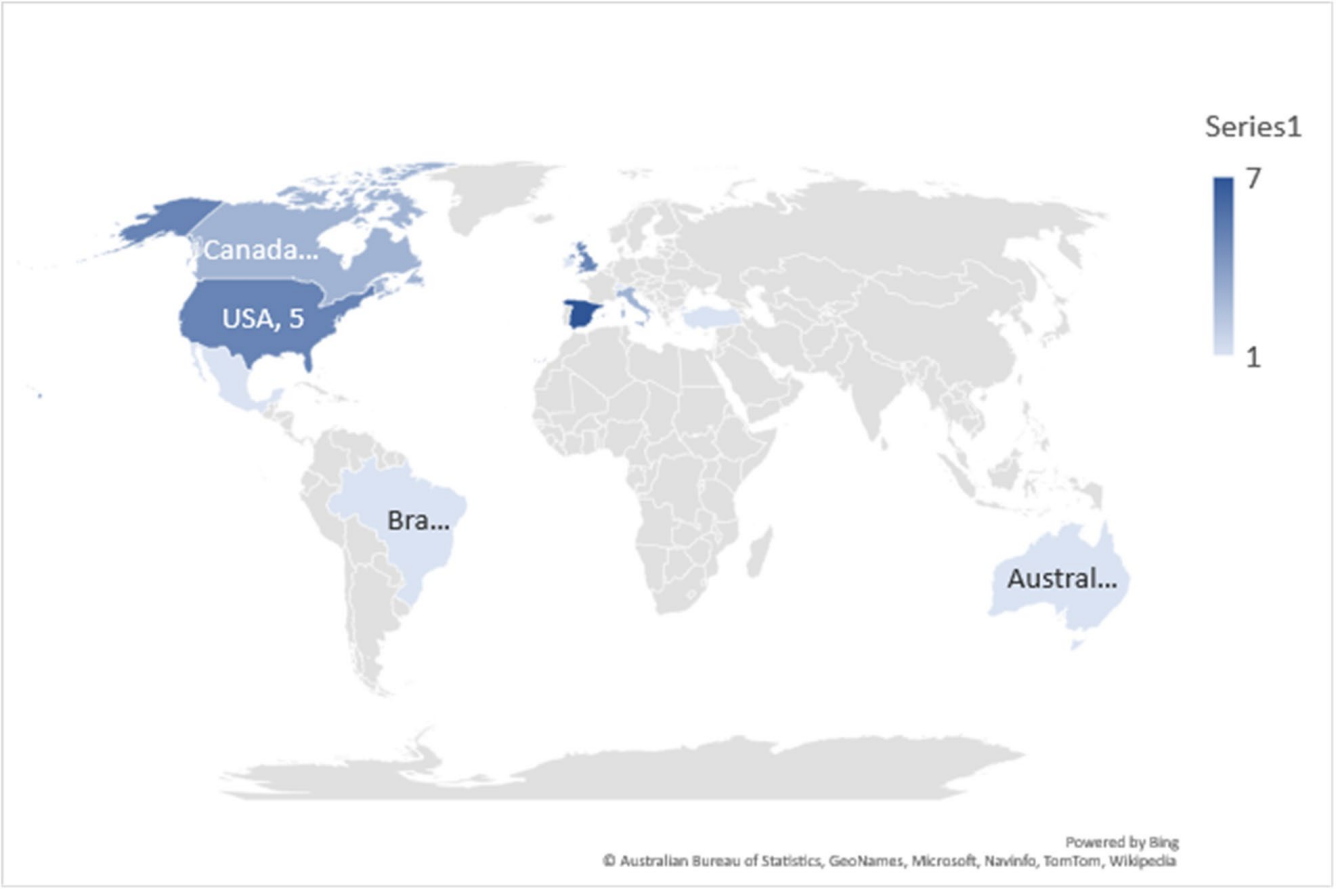
Map showing the distribution of studies included in the review. (Spain = 7; Italy = 3; Ireland = 1; USA = 5; Turkey = 1; Canada = 3: UK = 5; Mexico = 1; Australia = 1; Brazil = 1; Switzerland = 1)

Figure 1 gives a PRISMA chart of result of our search strategy and studies included in this synthesis. We identified a total of *N* = 31 studies [2, 6, 8, 11–13, 15, 18–41] across 4 continents (Fig. 2) that fulfilled the criteria for inclusion in the systematic review.

The total number of patients with polypharmacy as defined by the included studies was (N = 53347). The mean age of the patient cohort was 49.5 (SD ± 17.0) years, a significant proportion of which were males (67%). There was a total of 36 definitions of polypharmacy, with studies further divided based on the magnitude of polypharmacy (Table 1); minor polypharmacy (N = 3); major polypharmacy (N = 29); “severe” polypharmacy (N = 2); “excessive” polypharmacy (N = 1); “higher” polypharmacy (N = 1) (Table 1).

**Table 1:** Baseline and socio-demographic characteristics of the reviewed studies

Author	Study location	Study design	Duration of HIV (years)	HIV viral load (copies/ml)	Sample size (N)	PLWH	Age mean	Proportion of male population	Number of medications	Numerical definition	Duration of polypharmacy	Descriptive (HIV, non-HIV, or both)
Cantudo-Cuenca 2014	Spain	Prospective observational		UD (70.4%)	594	118	47	0.801	≥ 5	Yes		Non-HIV
Gimeno-Gracia 2015	Spain	Retrospective	13.6	UD (88%)	118	27	54.8	77.1	≥ 5	Yes	> 1 day, > 90 days, > 180 days	Both
Gimeno-Gracia 2016	Spain	Retrospective (descriptive)		UD (91.9%)	199	53	52	73.4	≥ 5	Yes	> 1 day, > 90 days, > 180 days	Non-HIV
Guaraldi 2016	Italy	Cross sectional	19		2944	992	48.37	66.3	≥ 5	Yes	4 months	Non-HIV
Guaraldi 2018	Italy	Cross sectional			1573	1258	71.47	82.7	≥ 5	Yes		Non-HIV
Halloran 2019	Ireland	Prospective observational			1072	158	56		≥ 5	Yes		Both
Holtzman 2013	USA	Cross sectional			3810	3810	44	79	≥ 5	Yes		both
Justice 2018	USA	Prospective observational			49,285	9473		97.5	< 5 and ≥ 2	Yes	90	Non-HIV
Kara 2019	Turkey	Cross sectional		145 (80.1%)	181	37	40.4 mean	79.6	≥ 5	Yes		Both
Krentz 2016	Canada	Prospective observational			1329	1329		75.8	≥ 5	Yes		Both
Lopes 2019	UK	Cross sectional			2680	2680	46 mean	86	≥ 5	Yes		Non-HIV
Lopez-Centeno 2019	Spain	Cross sectional			6636451	22945	48 (PLWH), 41 (control)	78.28. (PLWH), 48.02 (Controls)	≥ 5	Yes		Non-HIV
Mata-Marín 2019	Mexico	Case control	8	41–48 (63–80%)	125	125			≥ 5	Yes	0	Non-HIV
Mazzitelli 2019	UK	Prospective observational		UD	790	790	55.8	92.8	≥ 10, ≥ 5	Yes	0	Non-HIV
Morillo-Verdugo 2019	Spain	Cross sectional		184 (84.4)	223	223	53	86.5	≥ 6, ≥ 11, ≥ 21	Yes		Both
Nozza 2017	Italy	Cross sectional	17	UD (92%)	1222	1222	70	83.7	≥ 5	Yes	121 days	Non-HIV
Okoli 2020	25 countries	Prospective (multicentre)			2112	2112		70.4	≥ 5	Yes	–	Both
Patel 2015	UK	Cross sectional			299	16	58 mean	94.6	≥ 5	Yes		Both
Siefried 2017	Australia	Prospective observational			522	522	50.8	94.6	≥ 5	Yes		Non-HIV
Ssonko 2018	Uganda	Cross sectional			411	239			≥ 4	Yes		Non-HIV
Titon 2021	USA	Case control	9.2	UD	156	52	60	38.5	≥ 5	Yes		Non-HIV
Vinuesa-Hernando 2021	Brazil	Prospective observational			30	30	71	73	≥ 5, ≥ 10	Yes		Both
Arant 2021	Spain	Prospective cohort			348	106		77.2 (Cohort 1) and 72.1 (Cohort 2)	≥ 5	Yes		Both
Ramos 2021	USA	Prospective observational			39	24	54.5	87.2	≥ 11	Yes		Both
Calcagno 2021		Cross sectional			2432	1158	PLWH: 51.6 Controls: 47.7	69.8 (PLWH), 68.8 (Control)	≥ 10	Yes		Non-HIV
Loste 2020	Italy	Cross sectional	10	UD	91	91	71.2	81.3	≥ 5	Yes		Non-HIV
Livio 2020	Spain	Retrospective	18	159 (91%)	175	175	78	71	≥ 5	Yes		Non-HIV
Kuznetsov 2021	UK	Prospective observational	11.3		150	150	38.3	66	≥ 5	Yes	Daily	Both
Allemann 2017	Canada	Prospective observational			2	2	48	50	≥ 3	Yes	4 months	
Ware 2019	Switzerland	Prospective observational	12.5	UD (57.7%)	3160	1715	53	100	≥ 5	Yes		Non-HIV
Ware 2016	USA	Prospective observational	12	UD (48.2%)	3160	1715	46	100	≥ 5	Yes		Non-HIV

*UD* undetected HIV viral load

### Numerical definition of polypharmacy in PLWHA

There was marked variation in the numerical definition of polypharmacy, with studies reporting it as “minor”, “major”, “excessive”, “severe”, and “higher”. Of the studies included in our review, 93.5% (N = 29) defined polypharmacy as the concomitant use of greater than or equal to 5 medications. Several studies had “outlying” definitions for polypharmacy that were different from those mentioned in other studies. These definitions included ≥ 2 [12]; ≥ 3 [12]; ≥ 4 [13]; ≥ 6 [11, 26]; ≥ 10 [27, 40]; ≥ 11 [11, 26]; ≥ 21 [11].

### Duration of polypharmacy

Amongst the included studies, 16% (N = 5) incorporated the duration of treatment to the definition of polypharmacy, while a significant proportion 84% (N = 26) only provided the numerical definition of polypharmacy with no additional information on its duration. Gimeno-Gracia et al. further stratified drug exposure based on the following duration: greater than 1 day; greater than 90 days; and greater than 180 days [14, 37]. Three studies included a duration of more than or equal to 4 consecutive months [18, 21, 31]. In their definition of polypharmacy, Justice et al. included the duration of more than or equal to 90 consecutive days [12].

### ART or non-ART medication polypharmacy

With regards to constituents of medication regimen included in the definitions, 67.7% of studies (N = 21) specified that the polypharmacy definition included only non-ART medications, while 27% (N = 9) studies included both ART and non-ART medications in their definitions. One study did not specify whether ART and non-ART medications were used as part of the adjudication process of polypharmacy (3.2%) [24].

## Discussion

To our knowledge, this review represents the first comprehensive systematic synthesis of studies that have explored polypharmacy in PLWHA to define what the term polypharmacy entails in this context. We have identified and evaluated 31 studies that defined polypharmacy in PLWHA forming a pooled sample size of 53,347 from eleven countries. We found wide variability in the way polypharmacy was defined amongst PLWHA; with an iteration of a total of 36 definitions from the reviewed studies. We found a significant proportion (93.5%) of studies included in our report defined polypharmacy as ≥ 5 concomitant medications. Additionally, up to 67.7% have explicitly specified these concomitant medications as non-ART medications. Following a systematic synthesis of the results from all studies, we found that a definition of polypharmacy including ≥ 5 non-ART medications over any period is representative of what most studies put forth as their definition.

Several recent studies have identified HIV polypharmacy as a growing problem that needs to be addressed. However, the lack of consensus around a unified definition hindered attempts at exactly estimating the burden as well as robust appraisal of its consequences and interventions to mitigate its effect on therapeutics. Without adequately defining what constitutes polypharmacy, researchers, administrators, and clinicians are all liable to grossly misidentifying patients who are at risk for polypharmacy. This leaves patients susceptible to the dangerous (but avoidable) harms associated with polypharmacy including problematic interactions (drug–drug, drug–food, and pharmacogenetic), adverse effects, rising therapeutic costs, medication non-concordance, increased hospitalizations, and sometimes avoidable mortality [2]. The situation is especially serious in PLWHA since they are a high-risk therapeutic population ab initio with a high pill burden due to their rigid ART medication regimes. What has exacerbated this in recent years amongst these patient cohorts, is their increasing survival often associated with a tandem rise in prevalence of associated comorbidities [12, 22, 37, 42, 43]. Taken together, this provides the enabling milieu for potentially harmful polypharmacy to ensue often with far reaching implication for a range of morbidities as highlighted above.

Having a unified definition for polypharmacy ensures that at hospital, community, and administrative levels therapeutic decisions regarding the pill burden of PLWHA are more nuanced and uniform. Additionally, a well-established definition can also assist in strengthening and clarifying communication between different stakeholders to for example help encourage mindfulness and attention while prescribing medications, and minimize downstream adverse outcomes in these cohorts of patients. Finally, having a standardized definition for polypharmacy will allow a more robust comparison of parameters and outcomes related to pill burden in PLWHA and hence draw more precise conclusions.

Although most studies (93.5%) defined polypharmacy as the intake of 5 or more medications, there were several “outlier” studies that explored alternative definitions. The “outlier” definitions ranged from as low as ‘ ≥ 2 medications’ to as high as ‘ > 21 medications’, and it wasn’t immediately apparent from reviewing these studies how these thresholds were arrived at. From the average pill count of PLWHA starting at 3 medications, it is unlikely that adoption of polypharmacy thresholds < 5 in these patients is likely to be of any determinative value as literally all population of PLWHA will thus be classified as “cases”. Conversely, definitions at the upper part of the extreme (such as ≥ 21 medications) are likely misclassify a high proportion of PLWHA with high pill burden but which has not reached the high threshold of 21 medications. Yet other studies employed descriptive terms such as “excessive” [11] or “severe” (≥ 10 Medications) [24, 27], and “higher” [40] polypharmacy to convey the magnitude of pill burden, but these subcategories were not consistently mentioned across all studies.

Although, incorporating duration of drug exposure into the definition of polypharmacy allows for a better understanding of the magnitude of the problem, the time threshold it prescribes has the potential to preclude some patient cohorts from beneficial interventions simply because they fail to satisfy this arbitrary exposure thresholds. In our review, two studies included the duration of at least 4 months in their definition of polypharmacy (Nozza et al., Guaraldi et al.) [18, 21] while most other studies did not specify a duration. A patient taking several medications for 3 months may not be classified as a polypharmacy case according to these two studies but may be considered a polypharmacy case according to studies that did not include a duration of drug exposure in their definition. This has serious implications when it comes to deciding which patients will benefit from interventions aimed at reducing the harms caused by polypharmacy. If one definition of polypharmacy required a duration of more than 4 months, patient A who took 6 medications for 5 months would be offered interventions while patient B who took 9 medications for 2 months would not be a candidate for the same interventions (although both patients may be exposed to the same adverse outcomes of polypharmacy); including drug-drug, drug-food, and pharmacogenetic interactions amongst others. If a patient met the numerical criteria of polypharmacy but did not meet the criteria in terms of duration, they should not be excluded from interventions that address the effects of polypharmacy. The decision about whether a patient will benefit from interventions should be solely based on the adverse effects they are experiencing secondary to polypharmacy.

When incorporating duration as part of the polypharmacy definition, and when this same definition is used as criteria to guide interventions, it is important to consider both the number of medications and the duration of use simultaneously. If a patient has been using medications for a long time, fewer number of medications should be acceptable to meet the criteria for polypharmacy. Alternatively, if a patient has been using an excessively large number of medications but for a short period of time, interpretation of the duration of medication exposure should be more nuanced. In the general population for instance, before Masnoon et al.’s consensus review [10], Nishtala et al. [44] defined polypharmacy as the use of five to nine medications for 90 days or more, while Veehof et al. [45] described it as the ingestion of two or more medications for more than 240 days in a year. Although the duration was longer in the definition proposed by Veehof et al., the threshold for number of medications was lower in Nishtala et al.

Studies may agree with regards to the duration of medication exposure, but still diverge with regards to the numerical definition of polypharmacy. In patients with PLWHA, it is our observation that consistency amongst studies in only one component of polypharmacy definition (e.g. duration) is not enough. For instance, Nozza et al. [21], Guaraldi et al. [18], and Alleman et al. [31] all included a duration of 4 months in their definitions, different medication thresholds for what constitutes polypharmacy. More specifically, Alleman et al. considers ≥ 3 medications as polypharmacy while Nozza et al. and Guaraldi et al. considered ≥ 5 medications. According to the polypharmacy definition proposed by Alleman et al., a patient taking 4 medications for a duration of 5 months would fall under the definition of polypharmacy and may be eligible for interventions, but the same patient may not be offered interventions according to Nozza et al. or Guaraldi et al. This highlights the importance of having consistency in terms of both number of medications as well as duration of treatment.

While the majority (67.7%) of studies included in our review only comprised non-ART medications in their definitions of polypharmacy, a significant proportion (27%) embraced both ART and non-ART medications. Kara et al. [19] included both categories in their definition of polypharmacy, while Halloran et al. [6] only involved non-ART; although both studies had the same numerical definition for polypharmacy (5 or more medications). A patient taking three ART and three non-ART medications will meet the criteria for polypharmacy according to Kara et al. but not Halloran et al. Consistency with regards to the classes of medications included in the definition of polypharmacy is essential. The argument for inclusion of both ART and non-ART medications in the numerical count of polypharmacy is not difficult to discern. Inevitably ART drugs are increasingly associated with secondary therapeutic morbidities such as dyslipidemias, various kidney morbidity phenotypes (amongst others) and do often require additional and increasing number of medications (such as statins) to manage them. Therefore, although the latter class of drugs may not in the strictest sense of word be ART’s, but they constitute a package of inevitable therapeutics associated with PLWHA. Additionally, bidirectional and pharmacogenetic interactions highlighted earlier between ART and non-ART drugs have become an integral and sometimes unavoidable part of ART therapeutics (especially of they are not treatment-limiting). And in risk stratification of therapeutic burden such as polypharmacy in PLWHA, any attempt at excluding non-ART drugs from the definition has the potential to underestimate this risks/burden. Indeed, Justice et al.’s report did showed that more than two non-ART polypharmacy was associated with about 68% increased risk of mortality amongst PLWHA (hazard ratio 1.68, 95% CI 1.50–1.89). The novelty of Justice et al. ‘s study has been the first report to adjust for severity of illness to ascertain the exact contribution of both ART and non-ART related polypharmacy to morbidity and mortality outcomes in PLWHA population. In the light of these, and other factors exhaustively “ventilated” elsewhere, with regards to PLWHA, we recommend that both ART and non-ART medications be included in the definition of polypharmacy.

Despite the stated observations regarding the increasing risk of polypharmacy with rising survival of PLWHA, recent advances in drug delivery systems in these patients cohorts may likely alter our projection of polypharmacy risks. Cabotegravir and Rilpivirine (long-acting antiretroviral combination) both of which have received market authorization from European Medicines Agency (EMEA), and Food and Drug Administration (FDA) amongst other drug regulatory agencies look set to revolutionize the pill count per PLWHA [46, 47]. For example, a hitherto sustained pill burden of about 365 days may ultimately be reduced to just about 6 intramuscular injections per year. Further advances (such as the novel Islatravir (a first-in-class nucleoside reverse transcriptase translocation inhibitor) currently formulated as an implant with a dosing interval of about 1 year is speculated to significantly change this polypharmacy numerical dynamic going forward [48, 49]. Numerically the effect of this in the pill count per PLWHA will be considerably low, but what effect this will have on other downstream consequences of polypharmacy (bidirectional interactions) remains unknown and will need to be explored by future studies.

### Strengths and limitations

The key strength of this study lies in its novelty as the first attempt at unifying the various interactions of polypharmacy definitions in PLWHA currently in use in existing literature; thus providing therapeutic policy makers, researchers, and clinicians with an important tool to classify PLWHA in the context of polypharmacy. Furthermore, our methodology anchored on a robust systematic synthesis of current evidence from a plurality of studies contrast significantly from previous attempts that expounded a narrative inference to resolving this uncertainty. Finally, due to its loose inclusion criteria (both observational cohort studies and randomized controlled trials), this has allowed us to analyze a large, pooled sample size. This lends external validity to the study and ensures that the results are generalizable to a greater and more diverse patient population.

As has been observed from previous exploration of these data schemes, our study was limited by the same constraints; including missing data that sometimes could not be retrieved from some of the authors of the included studies; large variability and imprecise data points that may skew our results. Additionally, we have only included studies that were published in English language, it is however unlikely that the range of uncaptured data from studies in other languages are likely to alter the final point estimate of polypharmacy definition in any meaningful way. Future reviews will improve our understanding of the burden of polypharmacy in PLWHA by including the type of medications predominantly accounting for the excess numerical count as well as their duration of exposure. Despite this, the findings of this review are likely to engender a more robust and reproducible consensus around this rising morbidity.

## Conclusion

A plurality of studies in PLWHA have established that polypharmacy in this cohort of patients is the intake of ≥ 5 medications (both ART and non-ART). We recommend the incorporation of this definition into national and international PLWHA treatment guidelines to standardize the approach to addressing this rising morbidity.

## Supplementary Information

**Additional file 1: Table S1.** Results of rias of bias assessment of the included studies. Studies. **Table S2. **Review search strategy.

## Data Availability

All data relating to this work is available from the corresponding author on reasonable request.
