# Supplementary material for: What is polypharmacy in people living with HIV/AIDS? A systematic review
Source: AIDS Res Ther. 2022 Aug 2;19:37. doi: 10.1186/s12981-022-00461-4 (PMC9344688; doi:10.1186/s12981-022-00461-4)
Supplement: Supplementary file 1 — Additional file 1: Table S1. Results of rias of bias assessment of the included studies. Studies. Table S2. Review search strategy. [file 12981_2022_461_MOESM1_ESM.docx]

| Reviewed Studies | Selection bias |  | Performance bias | Detection Bias | Attrition Bias | Reporting Bias | Other Bias | TOTAL |
| --- | --- | --- | --- | --- | --- | --- | --- | --- |
|  | Random sequence generation | Allocation concealment | Blinding of participants and personnel | Blinding of outcome assessment | Incomplete outcome data | Selective reporting | |  |
| Cantudo-Cuenca ‎2014 | high | low | high | low | low | low | low | 2/7 |
| Gimeno-Gracia 2015 | low | low | high | high | low | low | low | 2/7 |
| Gimeno-Gracia 2016 | high | high | high | low | low | low | low | 3/7 |
| Halloran 2019 | high | low | low | low | low | low | low | 1/7 |
| Holtzman 2013 | high | low | high | high | low | low | low | 3/7 |
| Justice 2018 | low | low | low | low | low | low | low | 0/7 |
| Kara 2019 | high | high | high | low | low | low | low | 3/7 |
| Krentz 2016 | high | low | low | low | low | low | low | 1/7 |
| Lopes 2019 | high | low | low | low | low | low | low | 1/7 |
| Lopez-Centeno 2019 | low | low | high | low | low | low | low | 1/7 |
| Mata‑Marín 2019 | low | low | low | low | low | low | low | 0/7 |
| Mazzitelli 2019 | high | high | low | low | high | low | low | 2/7 |
| Morillo-Verdugo 2019 | high | high | high | low | high | high | low | 5/7 |
| Nozza 2017 | high | high | low | low | low | low | low | 2/7 |
| Okoli 2020 | low | low | low | low | low | high | low | 1/7 |
| Patel 2015 | low | low | low | low | low | low | low | 0/7 |
| Siefried 2017 | high | high | low | low | low | low | low | 2/7 |
| Ssonko 2018 | high | low | low | low | low | low | low | 1/7 |
| Titon 2021 | high | high | high | high | low | low | low | 4/7 |
| Vinuesa-Hernando 2021 | high | low | low | low | low | low | low | 1/7 |
| Arant 2021 | high | high | high | low | low | low | low | 3/7 |
| Ramos 2021 | high | high | low | low | low | low | low | 2/7 |
| Calcagno 2021 | low | high | high | low | low | low | low | 2/7 |
| Loste 2020 | high | low | low | low | low | low | low | 1/7 |
| Livio 2020 | high | low | low | low | low | low | low | 1/7 |
| Kuznetsov 2021 | high | high | high | low | low | low | high | 4/7 |
| Allemann 2017 | high | high | high | low | low | low | low | 3/7 |
| Ware 2019 | high | low | low | low | low | low | low | 1/7 |
| Ware 2016 | high | high | low | low | low | low | low | 2/7 |
| Guaraldi 2018 | high | low | low | low | low | low | low | 1/7 |
| Guaraldi 2017 | low | low | high | high | low | low | low | 1/7 |

**Additional file 1: TableS1.** Results of rias of bias assessment of the included studies. Studies

**Additional file 1: TableS2.** **Table: Review search strategy**

| HIV |
| --- |
| PLWH |
| People living with HIV |
| PLWHA |
| People living with HIV/AIDS |
| HIV (tiab) |
| 1 OR 2 OR 3 |
| Polypharmacy (tiab) |
| (((HIV) OR (PLWH)) OR (PLWHA)) |
| (((HIV) OR (PLWH)) OR (PLWHA)) AND (POLYPHARMACY) |
|  |
